# Adjuvant hyperbaric oxygen therapy reduces the duration of sporotrichosis treatment

**DOI:** 10.1371/journal.pntd.0013659

**Published:** 2025-10-29

**Authors:** Helena Duani, Alexandre Soares Bifano, Frederico Figueiredo Amâncio, Shinfay Maximilian Liu, Lilian Mesquita Gomes, César Macieira, Túlio Pinho Navarro, Unai Tupinambás

**Affiliations:** 1 Serviço de Infectologia, Departamento de Clínica Médica, Universidade Federal de Minas Gerais – UFMG, Belo Horizonte, Brasil; 2 Programa de Pós-graduação em Ciências da Saúde, Infectologia e Medicina Tropical, Faculdade de Medicina, Belo Horizonte, Brasil; 3 Unidade de Terapia Intensiva, Hospital das Clínicas, Universidade Federal de Minas Gerais – UFMG, Belo Horizonte, Brasil; 4 Unidade Laboratório de Análises Clínicas, Hospital das Clínicas, Universidade Federal de Minas Gerais – UFMG, Belo Horizonte, Brasil; 5 Núcleo de Educação em Saúde Coletiva, Universidade Federal de Minas Gerais, Belo Horizonte, Brasil; 6 Departamento de Cirurgia, Faculdade de Medicina, Universidade Federal de Minas Gerais – UFMG, Belo Horizonte, Brasil; 7 Programa de Pós-graduação Ciências Aplicadas à Cirurgia e à Oftalmologia, Faculdade de Medicina, Universidade Federal de Minas Gerais – UFMG, Belo Horizonte, Brasil; University of Florida, UNITED STATES OF AMERICA

## Abstract

**Background:**

Sporotrichosis is a subcutaneous mycosis caused by *Sporothrix spp.*, typically acquired through traumatic inoculation of fungal spores from soil, plants and infected cat scratches. The disease manifests in various clinical forms, including lymphocutaneous, ocular, bone, and disseminated infections. Standard treatment relies on prolonged administration of antifungals such as itraconazole, terbinafine, and potassium iodide, often requiring months for complete resolution. Moreover, treatment challenges include slow clinical response, recurrence, and potential drug toxicity. Hyperbaric oxygen therapy has been investigated as a potential adjuvant for invasive fungal infections due to its antimicrobial properties, enhanced tissue oxygenation, and immunomodulatory effects, which may contribute to improved healing and therapeutic outcomes.

**Methods:**

Patients with a confirmed diagnosis of cutaneous/lymphocutaneous sporotrichosis were divided into two groups to receive either itraconazole or itraconazole plus hyperbaric oxygen therapy. The primary outcomes were time to cure and therapeutic response. The intervention group was assessed weekly through clinical parameters and photographic records, and the control group was assessed monthly.

**Results:**

Patients who received hyperbaric oxygen therapy experienced a much shorter healing time than those who did not. The average time until a cure was 208.53 days in the itraconazole group compared to 57.54 days in the itraconazole plus hyperbaric oxygen therapy group. Patients in the Hyperbaric oxygen therapy group received an average of 18.23 hyperbaric sessions and were 65 times more likely to achieve faster healing than those who did not. Notably, with each additional year of age, the chances of rapid healing decreased by 2%.

**Conclusion:**

Our findings suggest that adjuvant hyperbaric oxygen therapy significantly accelerates the healing of fixed cutaneous/lymphocutaneous sporotrichosis. The results also highlight hyperbaric oxygen therapy´s potential to enhance treatment efficacy and reduce the burden of prolonged antifungal therapy. These results support further investigation of this valuable adjunctive treatment in the management of sporotrichosis.

## Author summary

Sporotrichosis is a skin infection caused by a fungus found in soil, plants and infected cats. People usually catch it through small cuts, scrapes and infected cat scratches. The most common type of this disease is lymphocutaneous, which can take several months or even a year to fully heal. Therefore, long-term treatment with antifungal medications like itraconazole may cause several side effects. In this study, we explored whether adding hyperbaric oxygen therapy (HBOT), a treatment where patients breathe pure oxygen in a pressurized chamber, could help patients recover faster. We compared two groups of patients — one group received only itraconazole, and the other group received itraconazole plus HBOT. The results showed that patients who received HBOT got better much faster — on average, in about 58 days — compared to those who only took itraconazole, who needed more than 200 days to heal. Interestingly, as people get older, their chances of healing quickly decrease by about 2% each year. Our findings suggest that HBOT could be a helpful addition to standard treatment, speeding up recovery and reducing the risks linked to long-term use of antifungal drugs.

## Introduction

Sporotrichosis is a subcutaneous mycosis caused by *Sporothrix spp.,* typically acquired through contact of the fungus with lesioned skin or mucous membranes, scratches or bites from infected animals, most commonly cats, and injuries caused by thorns, straw, or wood splinters [1].

Brazil is considered the epicenter of zoonotic transmission, especially of sporotrichosis transmitted by cats [2]. The primary etiological agent identified in Brazil is *Sporothrix brasiliensis*, but cases involving *S. schenckii* and *S. lurei* have also been reported [2].

Sporotrichosis is considered a neglected tropical disease due to the difficulty in controlling zoonotic transmission, the limited resources and knowledge for diagnosis, and the socioeconomic impact on affected populations [2]. Notably, the areas most affected by sporotrichosis in Brazil are marked by environmental and infrastructural vulnerability [1]. In addition, until January 2025, there was no compulsory notification for human sporotrichosis in Brazil [3], which impaired understanding of the real disease burden. This scenario makes it difficult to offer resources and implement effective public health measures to diagnose and control the disease.

In the past 20 years, the metropolitan region of Rio de Janeiro has been Brazil’s most important endemic area [4], with many sporotrichosis cases reported [5]. Lately, new endemic regions have emerged, accompanied by outbreaks and the identification of new pathogenic species [6].

The disease is presented in different clinical forms, such as lymphocutaneous, fixed cutaneous, ocular, pulmonary, osteoarticular, meningeal, and disseminated [7]. The lymphocutaneous sporotrichosis is the most common type [7], affecting patients between 30 and 40 years old [1]. On the skin, sporotrichosis usually begins on a finger or hand as a small, painless nodule, which gradually grows and develops into an open sore or ulcer. The lesion grows along the lymphatic vessels, mainly affecting the limbs and face [7].

Sporotrichosis standard treatment relies on prolonged administration of antifungals such as itraconazole or terbinafine, often requiring months to a year for complete resolution. Therefore, treatment challenges include slow clinical response, recurrence, and potential drug toxicity [7]. Despite advances in antifungal therapy, some forms of sporotrichosis may exhibit resistance or intolerance to these medications, highlighting the need for alternative treatments [8].

HBOT is a therapeutic administration of 100% oxygen (O_2_) in a pressurized environment, significantly increasing the partial pressure of oxygen in the tissues [9]. This increases tissue oxygenation, reduces inflammation, stimulates angiogenesis, and may potentiate the effects of antifungal agents [9]. Additionally, HBOT can modulate the immune response by promoting phagocytosis and the release of anti-inflammatory mediators [10].

HBOT has been investigated as a potential adjuvant in the treatment of various medical conditions, such as persistent infections and chronic wounds [11], including invasive fungal infections [9]. For instance, HBOT has been shown to improve symptom relief and promote wound healing in inflammatory skin diseases such as pyoderma gangrenosum when used alongside corticosteroids and immunosuppressants [12]. Moreover, studies show that HBOT effectively improves wound healing, reduces tissue necrosis, reduces infection, and reduces the likelihood of long-term complications when used as adjunctive therapy for severe lower limb soft tissue injuries [13].

In hard-to-heal wounds, HBOT combined with negative pressure wound therapy demonstrated a better wound healing rate and healing time [14]. However, the use of HBOT for diabetic foot ulcers lacks strong clinical evidence of benefits [15]. Despite that, there have been reports of lower rates of major amputations, enhanced ulcer healing, and reductions in ulcer size and depth with HBOT treatment compared to standard care [16].

The role of HBOT in sporotrichosis remains largely unexplored. There is a lack of clinical evidence for its use despite its antimicrobial properties, enhanced tissue oxygenation, and immunomodulatory effects, which may contribute to improved healing and therapeutic outcomes [9].

In this context, there is a need to investigate the effectiveness of adjuvant HBOT treatment on sporotrichosis. Therefore, this work aimed to evaluate if hyperbaric oxygen therapy could improve lymphocutaneous sporotrichosis standard treatment, decreasing the time needed for patients to heal.

## Methods

### Ethics statement

This study was approved by the Research Ethics Committee of Federal University of Minas Gerais under the following CAAE numbers (Certificate of Presentation for Ethical Consideration): 81319924.9.0000.8095 and 76717223.4.0000.5149. Participation was voluntary, and written informed consent was obtained from each patient or their legal representative.

### Study design

**Fig 1:**
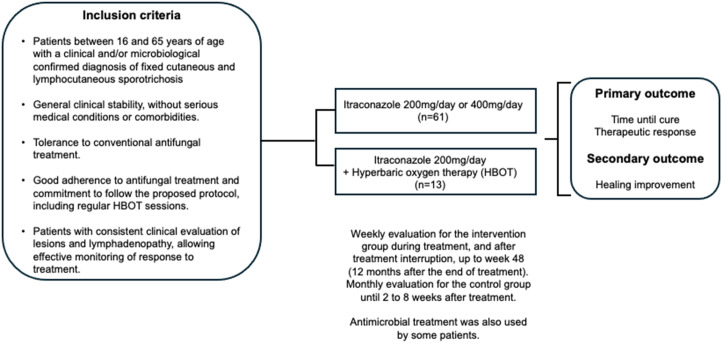
Study design.

A prospective study was conducted involving patients with a clinical and/or microbiological diagnosis of cutaneous/lymphocutaneous sporotrichosis treated by the infectious diseases department of the Manhuaçu Hyperbaric Clinic and the Infectious Diseases Clinics at Hospital das Clínicas from the Federal University of Minas Gerais (UFMG) between May 2021 and December 2024 (Fig 1).

Patients between 16 and 65 years of age, with a clinical and/or microbiological confirmed diagnosis of lymphocutaneous or fixed cutaneous sporotrichosis and general clinical stability, without severe medical conditions or comorbidities that may significantly interfere with response to treatment or participation in HBOT sessions, were included in the study. Other inclusion criteria were tolerance to conventional antifungal treatment, good adherence to antifungal therapy, and commitment to follow the proposed protocol, including regular HBOT sessions and adequate clinical evaluation of lesions and lymphadenopathy, allowing effective monitoring of treatment response.

Exclusion criteria included: 1) pregnancy and lactation; 2) uncontrolled chronic diseases such as diabetes mellitus, cardiac failure, or other medical conditions with increased risk associated with HBOT; 3) patients with conditions that prevent tolerance to HBOT, such as a history of pneumothorax, severe chronic obstructive pulmonary disease, or severe claustrophobia; 4) patients with severe immunosuppression, whether due to underlying medical conditions or the use of immunosuppressive medications; 5) patients with a documented history of significant adverse reactions to HBOT, such as otologic, sinus, or pulmonary barotrauma; 6) patients currently participating in other clinical studies to avoid interference with results or confusion in data analysis; 7) patients unable to adhere to treatment schedule, including regular HBOT sessions, which could compromise data consistency and integrity.

Candidates who meet the inclusion and exclusion criteria were selected to receive either standard antifungal treatment (200 or 400 mg/day itraconazole) or standard antifungal treatment (200mg/day itraconazole) combined with HBOT until lesion cure. Given that the hyperbaric chamber was situated in the Zona da Mata region, the majority of patients in this cohort were selected to undergo HBOT, primarily based on geographic proximity to their municipalities in the non-intervention group, the itraconazole dosage (200 mg or 400 mg daily) was determined according to clinical presentation. Patients with a higher number of lesions, larger or more extensive lesions, or those showing an initial poor response to therapy had their dose increased to 400 mg/day. Some patients in both groups also used antibiotic treatment during the study. Antibiotics were administered either when previously prescribed by attending physicians before the diagnosis of sporotrichosis, or by our team in cases with significant edema, erythema, and purulent discharge, suggesting possible bacterial superinfection. Patients were treated until complete improvement/healing of the lesion, which typically occurs within 4–24 weeks.

All clinical variables from both groups were collected and entered an anonymized database. The variables included sex, age, race, region of residence, microbiological culture results, route of infection, cat exposure and vaccination status, alcohol use, comorbidities, blood pressure, heart rate, clinical classification of sporotrichosis, site of involvement, initial clinical manifestations, laboratory test results, and antibiotic use.

### Hyperbaric oxygen therapy (HBOT)

The hyperbaric oxygen therapy (HBOT) protocol was standardized in accordance with international guidelines [17,18]. The therapeutic conditions were as follows: treatment pressure of 2.4 atmospheres absolute (ATA); session duration of 90 minutes; and administration of 100% oxygen, inhaled directly within the chamber pressurized environment, without the use of a face mask or hood. Oxygen flushing was performed in cases of thermal discomfort during the session to ensure patient safety and comfort.

Before the first session, patients underwent a clinical assessment to rule out absolute and relative contraindications to HBOT. During each session, clinical parameters were monitored, including blood pressure, heart rate and oxygen saturation to detect potential adverse events, including otalgia, barotrauma, and neurological symptoms indicative of oxygen toxicity. All patients received counseling on expected side effects, and the multidisciplinary care team was trained to promptly recognize and manage potential complications related to hyperbaric therapy.

All sessions were conducted in a monoplace hyperbaric chamber (ECOBAR 850 model, manufactured by OXY, Brazil), approved for human use by the Brazilian Health Regulatory Agency (Agência Nacional de Vigilância Sanitária – ANVISA; ANVISA registration no. 80009380005).

Patients underwent HBOT sessions three times per week, on non-consecutive days, allowing adequate intervals to promote tissue recovery while maintaining treatment adherence. The total duration of HBOT varied according to the clinical progression of each patient. The follow-up was conducted through clinical examinations and standardized photographic documentation (details are described in the next section).

HBOT discontinuation was guided by evidence of adequate wound healing, allowing individualized adjustments to optimize clinical outcomes. Discontinuation of HBOT was considered in the following situations: (i) complete remission of lesions, as determined by clinical evaluation; (ii) occurrence of serious adverse events contraindicating further treatment; (iii) patient non-adherence to the treatment protocol; and (iv) newly emerging contraindications to HBOT identified during clinical follow-up.

### Therapeutic response assessment

The primary outcome was time to cure, with weekly (for HBOT group) or monthly (for control group) evaluations of patient clinical progress. Patients in the HBOT group were evaluated weekly to monitor potential adverse effects and to accurately assess lesion progression. Patients in the non-intervention group were evaluated monthly, following the standardized clinical protocol in our service and national recommendations [7]. The therapeutic response was assessed using clinical parameters documented in medical records and research questionnaires, including the number and size of lesions, depth, nodulation, heat, edema, secretion, pain, pruritus, and infection progression. Additionally, photographic records were used to track lesion evolution every five HBOT sessions or earlier if substantial lesion improvement was observed. In the non-HBOT group, photographic documentation was performed monthly. Fungal culture growth was analyzed, and routine sporotrichosis consultation exams were also reviewed. After treatment completion, clinical progress was monitored every four weeks until week 48 based on medical record data. For the control group clinical progress was monitored every month until 2–8 weeks after treatment.

### Microbiological culture

Fungal cultures were performed by inoculating samples onto Mycosel and Sabouraud Dextrose agar, followed by incubation at 30°C for at least 30 days. Colonies suggestive of *Sporothrix spp*. initially appeared white to cream and moist, later developing a velvety surface and a darkened reverse that progressively became entirely black. Microscopic examination revealed thin, hyaline hyphae with slender conidiophores and small, oval to round conidia arranged in floral patterns, often resembling daisies or chrysanthemums.

Based on the initial clinical impression and the macroscopic characteristics of colony growth, combined with the observed micromorphology, it is often unnecessary to perform microculture of *Sporothrix spp*. for its identification.

### Statistical analysis

#### Sample size calculations.

The sample size calculation was performed using the software R (version 4.4.0), setting a 95% confidence level, 80% Power and Ratio of 3. This resulted in 52 patients that should be included in the study using the Fleiss sample calculation, being 13 patients in the HBOT group.

#### Data statistical analysis.

A longitudinal study design was employed to evaluate patients who did or did not undergo HBOT, aiming to investigate associations between demographic, clinical characteristics and patient outcomes over time. Categorical variables were summarized using absolute and relative frequencies, whereas continuous variables, including time to healing and the number of HBOT sessions, were described using measures of central tendency and dispersion. The normality of continuous variables was assessed using the Shapiro-Wilk test.

To compare groups and examine associations, the Chi-square test, Fisher’s exact test, and the Mann-Whitney test were applied, as appropriate. Time to healing was analyzed using survival analysis techniques. Initially, bivariate analyses were conducted using the Log-rank test for categorical predictors and parametric survival models—specifically Exponential, Weibull, and Log-Normal distributions—for continuous predictors. These models were compared to a null model (without covariates) using the likelihood ratio test. Variables showing potential association in the bivariate analysis were entered into a multivariate Cox proportional hazards regression model. Variable selection was performed using a stepwise approach, combining backward elimination and forward selection methods.

## Results

### Patient characteristics

Most patient characteristics were well-balanced between the two groups (Table 1). However, a higher proportion of patients in the itraconazole-only group were of Brown or mixed race and resided in Belo Horizonte. In contrast, patients in the itraconazole + HBOT group were predominantly White, Brown or mixed race and lived in the Zona da Mata region of Minas Gerais.

**Table 1:** Patient characteristics.

		Itraconazole only (n = 61)		Itraconazole + HBOT (n = 13)		P-value
		N	%	N	%	
Sex	Female	39	63.93%	7	53.85%	0.539
	Male	22	36.07%	6	46.15%	0.539
Age	< 60 years	43	70.49%	11	84.62%	0.493
	≥ 60 years	18	29.51%	2	15.38%	0.493
	Average age (SD)	51.39 (16.83)		46.23 (16.32)		0.317
Race	White	3	4.92%	6	46.15%	0.001
	Brown or mixed race	51	83.61%	7	53.85%	0.001
	Black	7	11.48%	0	0.00%	0.001
City of residence	Belo Horizonte	42	68.85%	1	7.69%	0.000
	Greater Belo Horizonte metropolitan area	18	29.51%	0	0.00%	0.000
	Zona da Mata of Minas Gerais	1	1.64%	12	92.31%	0.000
Microbiological culture	No	28	45.90%	5	38.46%	0.855
	Yes	33	54.10%	8	61.54%	0.855
Culture material	Not performed	28	45.90%	5	38.46%	0.006
	Biopsy	24	39.34%	1	7.69%	0.006
	Swab	9	14.75%	7	53.85%	0.006
Contact with soil, ground, or gardening	No	53	86.89%	6	46.15%	0.003
	Yes	8	13.11%	7	53.85%	0.003
Contact with cats	No	8	13.11%	0	0.00%	0.336
	Yes	53	86.89%	13	100.00%	0.336
Cat vaccinated against rabies	Unknown	50	81.97%	5	38.46%	0.000
	No	10	16.39%	1	7.69%	0.000
	Yes	1	1.64%	7	53.85%	0.000
Alcoholic beverages consume	No	51	83.61%	10	76.92%	0.688
	Yes	10	16.39%	3	23.08%	0.688
Previous illnesses/ comorbidities	No	14	22.95%	9	69.23%	0.022
	Diabetes Mellitus	3	4.92%	0	0.00%	0.022
	Hypertension	18	29.51%	0	0.00%	0.022
	Hypertension and Diabetes Mellitus	9	14.75%	0	0.00%	0.022
	Smoking	4	6.56%	0	0.00%	0.022
	More than 3 comorbidities	3	4.92%	1	7.69%	0.022
	Other	10	16.39%	3	23.08%	0.022
Systolic blood pressure (Average ± SD)		125.44 ± 16.27		125.92 ± 17.73		0.868
Diastolic blood pressure (Average ± SD)		78.58 ± 9.57		84.31 ± 17.12		0.367
Heart rate or pulse rate (Average ± SD)		76.04 ± 12.41		79.46 ± 10.24		0.316

More than 50% of patients in both groups had microbiological cultures performed through biopsy or lesion swab. In the itraconazole-only group, 86.89% of patients reported no contact with soil, ground, or gardening, while in the itraconazole + HBOT group, 53.85% reported soil contact. Notably, all patients in the HBOT group had contact with cats compared to 86.89% in the itraconazole-only group.

Regarding comorbidities, 69.23% of patients in the HBOT group reported no previous illnesses, and 7.69% had more than three comorbidities. In contrast, only 22.95% of patients in the itraconazole-only group had no comorbidities. Approximately 30% of all patients had hypertension.

### Clinical parameters

Lymphocutaneous sporotrichosis was the predominant form of the disease in both groups, affecting 70.49% of patients in the itraconazole-only group and 61.54% in the HBOT group. Most lesions were located on the upper limbs (Table 2).

**Table 2:** Clinical features.

		Itraconazole only (n = 61)		Itraconazole + HBOT (n = 13)		P-value
		N	%	N	%	
Patient’s clinical classification	Fixed cutaneous	18	29.51%	5	38.46%	0.526
	Lymphocutaneous	43	70.49%	8	61.54%	0.526
Site of involvement	More than four sites/segments	2	3.28%	0	0.00%	1.000
	Lower limbs	8	13.11%	2	15.38%	1.000
	Upper limbs	51	83.61%	11	84.62%	1.000
Fever	No	57	93.44%	13	100.00%	1.000
	Yes	4	6.56%	0	0.00%	1.000
Weight loss	No	56	91.80%	13	100.00%	0.579
	Yes	5	8.20%	0	0.00%	0.579
Cervical lymph nodes	No	61	100.00%	10	76.92%	0.004
	Yes	0	0.00%	3	23.08%	0.004
Axillary lymph nodes	No	50	81.97%	11	84.62%	1.000
	Yes	11	18.03%	2	15.38%	1.000
Lymph nodes in the arms	No	28	45.90%	8	61.54%	0.472
	Yes	33	54.10%	5	38.46%	0.472
Inguinal lymph nodes	No	60	98.36%	13	100.00%	1.000
	Yes	1	1.64%	0	0.00%	1.000
Edema of lower limbs	No	59	96.72%	13	100.00%	1.000
	Yes	2	3.28%	0	0.00%	1.000

Systemic symptoms such as fever, weight loss, and lymphadenopathy in the cervical, axillary, or inguinal regions were uncommon in both groups, as was lower limb edema. Conversely, lymph node enlargement in the arms was observed in 54% of patients in the itraconazole-only group and 38% in the HBOT group, likely due to the frequent involvement of upper limbs.

Routine sporotrichosis laboratory tests were also reviewed, and we did not observe any alteration or improvement on these measurements between the two groups (Table 3).

**Table 3:** Routine sporotrichosis laboratory tests.

		Itraconazole only (n = 61)	Itraconazole + HBOT (n = 13)	P-value
		Average ± SD	Average ± SD	
Aspartate Aminotransferase	first value	26.87** ±** 13.50	22.33** ± **8.14	0.531
	highest value	34.16** ± **31.89	23.60** ± **11.50	0.723
Alanine Aminotransferase	first value	26.87** ± **26.45	17.44** ± **7.07	0.105
	highest value	36.49** ± **47.25	21.60** ± **11.65	0.282
Alkaline Phosphatase	first value	104.33** ± **57.29	76.00** ± **21.86	0.251
	highest value	101.84** ± **52.67	67.67** ± **8.33	0.207
Gamma-Glutamyl Transferase	first value	54.29** ± **77.84	42.33** ± **47.11	0.406
	highest value	61.60** ± **79.75	25.75** ± **13.94	0.164
Total bilirubin	first value	0.56** ± **0.25	0.67** ± **0.47	0.492
	highest value	0.66** ± **0.28	0.69** ± **0.65	0.376
Direct bilirubin	first value	0.25** ± **0.24	0.33** ± **0.20	0.142
	highest value	0.24** ± **0.19	0.36** ± **0.29	0.358
Amylase	first value	68.59** ± **21.95	26.75** ± **52.83	0.211
	highest value	78.24** ± **24.21	19.00	0.100
Lipase	first value	62.98** ± **32.43	21.50** ± **3.54	0.000
	highest value	75.32** ± **41.29	106.00	0.281
Creatinine	first value	0.84** ± **0.21	1.05** ± **0.30	0.098
	highest value	0.86** ± **0.22	1.00	0.312
Hemoglobin (first value after treatment start)		14.07** ± **1.64	13.38** ± **1.89	0.342
Red blood cells (first value after treatment start)		4.50** ± **0.38	4.45** ± **0.47	0.524
Total white blood cell count (first value after treatment start)		7277.26 **± **2020.86	7768.56** ± **3516.93	0.987
Neutrophils (first value after treatment start)		4783.30** ± **1721.07	5156.56** ± **2811.34	0.855
Lymphocytes (first value after treatment start)		1527.38** ± **742.26	1874.89** ± **908.22	0.318
Eosinophils (first value after treatment start)		128.39** ± **103.22	181.43** ± **258.41	0.401
Platelets (first value after treatment start)		298793.10** ± **94246.40	232444.44** ± **38649.42	0.050

### Treatment response

A greater proportion of patients in the itraconazole-only group used additional antimicrobial medications (52.46%) compared to those in the HBOT group (38.46%). All patients in the HBOT group received itraconazole at a dose of 200 mg/day, while 13% of patients in the itraconazole-only group received a higher dose (400 mg/day) (Table 4).

**Table 4:** Antimicrobial Treatments.

		Itraconazole only (n = 61)		Itraconazole + HBOT (n = 13)		P-value
		N	%	N	%	
Treatment with antifungal medication	Itraconazole 200 mg/day	53	86.9%	13	100.00%	0.336
	Itraconazole 400 mg/day	8	13.11%	0	0.00%	0.336
Treatment with antimicrobial medication	No	29	47.54%	8	61.54%	0.442
	Amoxicillin	1	1.64%	1	7.69%	0.442
	Amoxicillin + Cephalexin	1	1.64%	0	0.00%	0.442
	Amoxicillin + Doxycycline	0	0.00%	1	7.69%	0.442
	Amoxicillin clavulanate	13	21.31%	2	15.38%	0.442
	Amoxicillin clavulanate + Azithromycin	3	4.92%	0	0.00%	0.442
	Amoxicillin clavulanate + Cephalexin	4	6.56%	0	0.00%	0.442
	Azithromycin	1	1.64%	0	0.00%	0.442
	Cephalexin	8	13.11%	1	7.69%	0.442
	Doxycycline	1	1.64%	0	0.00%	0.442
Duration of prescribed antimicrobial treatment (days - average ± SD)		4.72 ± 5.59		3.62 ± 5.36		0.430

**Fig 2:**
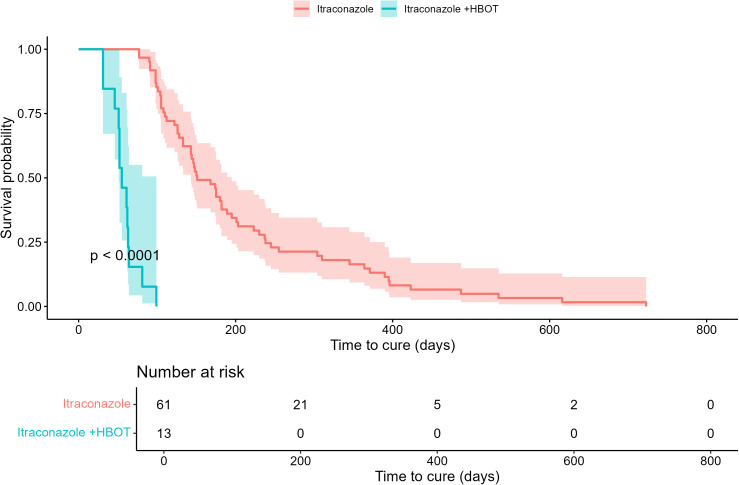
Kaplan-Meier curves for time to cure: a) comparing Itraconazole vs. Itraconazole + HBOT. Log-Rank test, p < 0.0001.

Patients who underwent HBOT experienced significantly shorter healing times (Table 5 and Fig 2). The average time to cure was 208.53 days in the itraconazole-only group versus 57.54 days in the itraconazole + HBOT group, indicating a 3.6-fold faster recovery. The average number of HBOT sessions was 18.23.

**Table 5:** Average time until cure and average number of HBOT sessions.

	Itraconazole only (n = 61)	Itraconazole + HBOT (n = 13)
**Average total treatment** **time until cure (days)**	208.53	57.54
**Average number of** **hyperbaric sessions**	0	18.23

**Fig 3:**
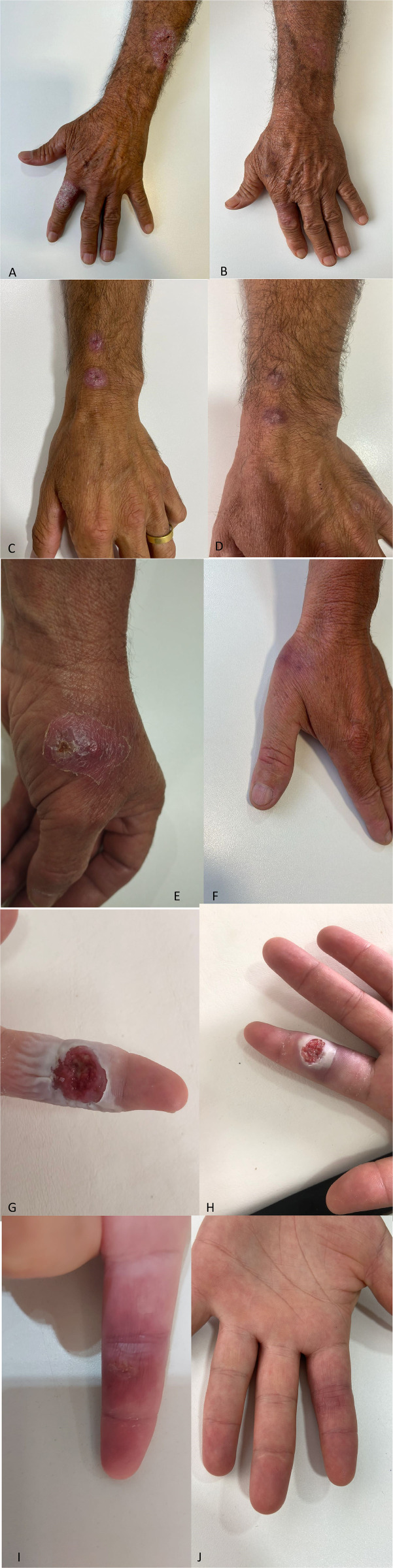
Photographic records monitoring treatment evolution and lesion healing over time on HBOT treatment group. Patient 1: A) Without treatment left arm and second finger. A) Day 27 after HBOT. Patient 2: C) Left wrist, day zero, without treatment. D) Left wrist, day 60 HBOT. Patient 3: E) Left hand day zero, without treatment. F) Left hand, day 27 HBOT. Patient 4: G) Left hand, second finger, day zero, with no treatment. H) Left hand, second finger, day one of HBOT and itraconazole. I) Left hand, second finger, day 14, HBOT and itraconazole. J) Left hand, second finger, day 30, HBOT and itraconazole.

Treatment response was documented through photographic records, which demonstrated lesion improvement or complete healing by approximately 60 in more severe cases and by around day 30 in more superficial lesions (Fig 3) in some patients of the HBOT group.

We compared pre-defined subgroups through multivariate analysis to assess the likelihood that HBOT improves healing time (Table 6). Firstly, patients receiving HBOT were 65 times more likely to achieve rapid healing than those who did not. Additionally, male patients were 1.6 times more likely to heal quickly than females. Each additional year of age was associated with a 2% decrease in the likelihood of rapid healing.

**Table 6:** Relative chances of rapid cure in pre-specified subgroups.

Subgroups	Hazard ratio (HR)	p-value
Hyperbaric oxygen therapy	65.97	6.21e^-13^
Male sex	1.62	0.0632
Age	0.98	0.0771
Clinical classification - Lymphocutaneous	1.86	0.0552
Lower limb involvement	0.36	0.2239
Upper limb involvement	0.21	0.041

## Discussion

This study assessed the impact of HBOT as an adjuvant treatment to itraconazole for sporotrichosis. The results demonstrated that HBOT in combination with itraconazole improved cutaneous and lymphocutaneous sporotrichosis treatment and decreased therapy duration (57.54 days for HBOT group vs. 208.53 days for itraconazole-only group). A multivariate analysis revealed that patients treated with HBOT were 65 times more likely to achieve faster healing. Male sex was also associated with a 1.6-fold increase in likelihood of faster recovery, whereas increasing age was negatively correlated with healing speed. The observed benefit of HBOT in accelerating wound healing aligns with its proposed mechanisms of action. The faster healing times observed with HBOT are consistent with findings in the management of severe lower limb soft tissue injuries [13] and diabetic foot ulcers, where HBOT has been investigated for its potential to enhance wound healing rates [15] and reduce amputation risks [16]. In the context of invasive fungal infections, for which itraconazole is often used, studies suggest a potential role for HBOT as an adjunctive therapy [19]. For instance, *in vitro* studies have shown that HBOT can inhibit fungal biofilm formation, such as with *Aspergillus fumigatus*, by elevating tissue oxygen levels and enhancing antifungal activity [19].Furthermore, in a murine model of invasive pulmonary aspergillosis, HBOT showed promise in slowing disease progression and extending survival, although robust fungal growth inhibition *in vivo* with once-daily treatment was not achieved [19]. Given the similarities in fungal pathogenesis and oxygen-deprived microenvironments, these findings may also be applicable to other deep fungal infections, such as sporotrichosis, as demonstrated in the present study.

Faster healing reduces the duration of antifungal therapy, lowers the risk of complications, and improves patient quality of life [9]. Additionally, the fact that all patients in the HBOT group received a standard itraconazole dose of 200 mg/day, while a subset of the itraconazole-only group required a higher dose (400 mg/day), suggests that HBOT may exert a synergistic effect with antifungal agents [20]. The reduced need for additional antimicrobials in the HBOT group is also noteworthy, especially given the increasing concern about antimicrobial resistance [21] and the potential to limit drug interactions and adverse effects.

This is particularly relevant for immunocompromised patients or those with comorbidities that impair wound healing [22], for whom single antifungal agents might not be sufficient. Comorbidities such as HIV infection, cardiovascular diseases, and diabetes are common among sporotrichosis patients and could worsen the disease progression [1]. Immunocompromised individuals, such as those with HIV/AIDS, solid organ transplant recipients, and patients undergoing long-term corticosteroid therapy, may develop severe forms of fungal infections, including sporotrichosis, and could benefit from this treatment combination [22]. Our findings may have broader applicability, particularly for patients with complicated or treatment-resistant sporotrichosis. It has been shown that patients’ refractory to conventional treatments for other infectious diseases, like zygomycosis, demonstrate clinical and microbiological improvements after the inclusion of HBOT in their therapeutic protocols [10].

A major factor in adopting HBOT for lymphocutaneous sporotrichosis is the treatment cost. Each HBOT session performed in the ECOBAR 850 single-person chamber has an approximate unit cost of R$ 1,738.68 (BRL) [23], which is equivalent to about $307.81 USD per session (based on the average exchange rate of 1 USD = 5.10 BRL (as of April 2025). During the study, patients in the HBOT group underwent an average of 18.23 sessions. Therefore, the average total cost of hyperbaric treatment per patient was approximately $5,611.37. Despite the high initial cost, data from the present study suggest that the investment may be offset by the clinical benefits observed, such as a marked reduction in healing time and decreased need for additional antimicrobials. These outcomes indicate a positive impact not only on individual recovery but also on the reduction of indirect healthcare system costs, including medical appointments, diagnostic tests, and hospitalizations. Indeed, HBOT reduced symptoms of radiation cystitis in cancer patients and decreased procedures and hospitalizations demonstrating cost-effectiveness when used together with routine interventions [24]. Similarly, it has been shown that HBOT is cost-effective compared with standard care in the treatment of diabetic foot ulcers [25]. Therefore, although HBOT has been incorporated into Brazil’s public health system (SUS) for a limited number of conditions, its implementation remains challenging and difficult to access. HBOT shows a promising incremental cost-effectiveness profile that supports its inclusion in clinical protocols, particularly if further large-scale pharmacoeconomic studies are conducted.

The main strength of our results is the magnitude of the effect observed. A 3.6-fold faster recovery time and a 65-fold increased likelihood of rapid healing in the HBOT group strongly suggest a significant therapeutic benefit. The finding that the HBOT group required fewer additional antimicrobial medications provides further support for the efficacy of HBOT as an adjuvant therapy, potentially indicating improved control of the infection.

Currently, there are no clinical studies evaluating HBOT in the treatment of sporotrichosis. Although case reports and small trials indicate promising results, general limitations include heterogeneity in study designs, patient characteristics, and HBOT protocols, such as pressure, duration and number of sessions, as well as lack of comprehensive long-term follow-up data [13]. These factors may restrict the generalizability of findings.

The sample size of our study is adequate considering the magnitude of the response in the HBOT group but may restrict generalizability and limit subgroup analyses. Moreover, potential for unmeasured confounders should be considered when interpreting the results. There could also be other confounding factors associated with a higher chance of recovery, such as treatment adherence and socioeconomic support.

The HBOT group was smaller in size, but its baseline characteristics were comparable to the itraconazole-only group. Both groups were predominantly female, most patients were younger than 60 years, the majority were of brown skin color, cat exposure was frequent, and no significant differences were observed in comorbidities. In the non-intervention group, itraconazole dosage was occasionally increased to 400 mg/day in patients with more extensive disease, characterized by a larger number or size of lesions, or in those who failed to show adequate improvement with the standard 200 mg/day dose. This clinical practice is common and consistent with the literature, where itraconazole regimens for sporotrichosis range from 100 mg to 400 mg/day, and the optimal dose for specific subgroups has not been definitively established. Moreover, a previous study has shown that the efficacy and safety of itraconazole pulse therapy are comparable to those of the continuous regimen [26], further supporting flexibility in dosing strategies. All patients were followed for at least one to eight months after completion of treatment, depending on individual clinical characteristics. In some cases, patients returned for additional evaluations, either for unrelated reasons or due to concern about possible relapses of their lesions. However, no cases of relapse were observed during the follow-up period in this study.

Some heterogeneity between the HBOT and non-HBOT groups, together with the small number of patients in the HBOT arm, may have influenced the results. In particular, patients in the HBOT group were evaluated more frequently and may have shown greater adherence compared to those treated with itraconazole alone. Nevertheless, potential confounding factors were controlled for, ensuring that the main clinical characteristics were comparable between groups. In addition, all patients had full access to the research team to clarify doubts and request consultations when needed. The identification of factors like male sex and younger age being associated with a higher likelihood of rapid healing in the HBOT group suggests that patient stratification might be important to optimize the use of this adjunctive therapy. Further research should explore these subgroup effects and identify features of HBOT responsiveness to determine which patient populations are most likely to benefit from the treatment.

Recent studies have demonstrated that different species within the *Sporothrix* genus exhibit significant variability in virulence. Among the pathogenic species, *Sporothrix brasiliensis* stands out as the most virulent, particularly in Brazil, where it has driven large zoonotic outbreaks. Experimental models show that *S. brasiliensis* causes higher fungal burden, severe tissue damage, and increased mortality compared to *S. schenckii* and *S. globosa* [27,28]. Clinically, infections by *S. brasiliensis* are associated with more severe and atypical presentations, including disseminated and extracutaneous forms, even in immunocompetent individuals [29]. Its enhanced thermotolerance, melanin production, and efficient cat-to-human transmission contribute to its epidemiological success [27].

In our study, we did not perform species differentiation or genotyping; however, considering the epidemiological context and previous molecular studies in Minas Gerais, it is highly probable that the isolates belong to *S. brasiliensis*, which predominates in this region. One study provides clear evidence of the predominance of a genetic group profile circulating in animals in Minas Gerais, independent of that disseminated from Rio de Janeiro. [30].

There is a clinical rationale for using HBOT in sporotrichosis, based on the observation that drug delivery is impaired in poorly vascularized or necrotic tissues, such as sporotrichosis lesions [31]. However, HBOT is not officially recognized in current treatment guidelines, which remain focused on antifungal therapies [7]. The Brazilian Society of Dermatology recommends thermotherapy, cryosurgery, and electrosurgery as adjuvant treatment options [7]. It also states that there have been reports of successful use of photodynamic therapy, either alone or in combination with intermittent doses of itraconazole [7].

Therefore, we must acknowledge the need for more robust, controlled studies to validate our observed results. Mechanistic investigations will be crucial to understand the underlying synergy between HBOT and antifungal agents such as itraconazole. Moreover, randomized controlled trials on the efficacy of HBOT in sporotrichosis and other fungal infections should investigate how different HBOT protocols, such as pressure levels, session duration, and frequency, affect fungal dissemination and cure mechanisms. This approach will help to determine HBOT value as a routine component of sporotrichosis treatment.

## Conclusion

Our results support the potential of HBOT as an effective combination to itraconazole in the treatment of lymphocutaneous sporotrichosis. HBOT promoted faster healing and reduced the need for additional antimicrobial agents. While further randomized trials are needed, this study adds to the growing evidence that HBOT may enhance outcomes in infections marked by impaired healing or antifungal resistance.

## Supporting information

S1 Data Excel table with all raw data required to replicate the results of this study. (XLSX)
